# Preparation of Cholesterol-Modified Hyaluronic Acid Nanogel-Based Hydrogel and the Inflammatory Evaluation Using Macrophage-like Cells

**DOI:** 10.3390/gels9110866

**Published:** 2023-10-31

**Authors:** Kohei Yabuuchi, Mika Suzuki, Chen Liang, Yoshihide Hashimoto, Tsuyoshi Kimura, Kazunari Akiyoshi, Akio Kishida

**Affiliations:** 1 New Product Development Office, R&D Group, Healthcare Materials Division, Life Innovation SBU, Asahi Kasei Co., Chiyoda-ku, Tokyo 100-0006, Japan; 2 Institute of Biomaterials and Bioengineering, Tokyo Medical and Dental University, 2-3-10 Kanda-surugadai, Chiyoda-ku, Tokyo 101-0062, Japan; 3 Department of Immunology, Graduate School of Medicine, Kyoto University, Yoshida-Konoe-cho, Sakyo-ku, Kyoto 606-8501, Japan

**Keywords:** hydrogel, nanogel, hyaluronic acid, THP-1, pro-inflammation

## Abstract

Nanogels are candidate biomaterials for tissue engineering and drug delivery. In the present study, a cholesterol–hyaluronic acid hydrogel was developed, and the pro-inflammatory response of macrophages to the hydrogel was investigated to determine its use in biomedical applications. Hyaluronic acid modified with cholesterol (modification rate: 0–15%) and maleimide (Chol-HA) was synthesized. The Chol-HA nanogel was formed through self-assembly via hydrophobic cholesterol interactions in aqueous solution. The Chol-HA hydrogel was formed through chemical crosslinking of the Chol-HA nanogel via a Michael addition reaction between the maleimide and thiol groups of 4arm−PEGSH. We found that the Chol-HA hydrogels with 5, 10, and 15% cholesterol inhibited the pro-inflammatory response of HiBiT−THP−1 cells, suggesting that the cholesterol contributed to the macrophage response. Furthermore, Interleukin 4 (IL−4) encapsulated in the hydrogel of the Chol-HA nanogel enhanced the inhibition of the inflammatory response in HiBiT-THP-1 cells. These results provide useful insights into the biomedical applications of hydrogels.

## 1. Introduction

Hyaluronic acid (HA, also known as hyaluronan), is an anionic, non-sulfated glycosaminoglycan composed of repeating disaccharide units, β-1,4-D-glucuronic acid, and β-1,3-N-acetyl-D-glucosamide [1]. HA is a critical component of the extracellular matrix and plays an important role in cellular processes. HA has immunomodulatory effects: low-molecular-weight HA stimulates M1-like macrophage activation (pro-inflammatory) and nitric oxide release, and high-molecular-weight HA stimulates M2-like macrophage activation (anti-inflammatory) as well as Arg1 and IL10 upregulation [2]. Several drug delivery systems (DDS) [3,4,5,6] and hydrogels [7,8,9,10,11] use HA-based hydrogel materials. Since HA hydrogel stiffness is associated with CD44-mediated mechanosensitive signaling, crosslinked HA can effectively regulate cell adhesion and migration [12]. In addition, HA is an ideal medical biomaterial owing to its low toxicity as well as biocompatibility with the human body and angiogenesis [13]. HA is also useful in orthopedics and ophthalmology and has been applied in clinical practice [14]. 

Nanogels are three-dimensional hydrogel materials in the nanoscale size range formed via the chemical and physical crosslinking of polymer chains. Akiyoshi et al. first reported the preparation of cholesterol-modified pullulan (CHP) nano gels via self-assembly via hydrophobic cholesterol moieties [15]. Owing to their ability to encapsulate proteins and nucleic acids, CHP nanogels have been applied in drug delivery [16]. Numerous substrates and methods have been employed in nanogel preparation [17,18]. 

Recently, nanogels with reactive moieties, such as acryloyl, carbonyl, amino, and thiol groups, have been synthesized, and nanogel-based hydrogels have been prepared by crosslinking reactive moieties [17]. Moreover, the presence of nanogel domains in hydrogels makes them useful scaffolds for tissue engineering because the nanogels are expected to function in a chaperone-like manner [19]; that is, the proteins are trapped in the nanogel without aggregation and released in their native form [15]. These scaffolds have been applied in bone and muscle regeneration [17]. Bone morphogenetic protein-2 (BMP-2), incorporated into CHP-crosslinked hydrogels, promotes new bone formation in mice with bone defects, through BMP-2 stimulation. Also, HA-based nanogels have been prepared by introducing cholesterol moieties [20]. In contrast to pullulan, the physical properties of HA-based nanogels can be modulated by increasing the degree of cholesterol substitution on the HA polysaccharide chain. By maintaining the cholesterol modifications within a specific range, the nanogels precipitate under physiological salt conditions, suggesting that they can serve as effective sustained DDS materials. Conversely, within a different range, stable nanoparticles are formed under physiological salt conditions, suggesting that they could be used in targeted delivery applications. Furthermore, they can improve the loading capacity of protein pharmaceuticals [20].

The temporal regulation of M1-like and M2-like macrophages is crucial for tissue regeneration. When using HA-based hydrogels, during early- and late-stage skeletal muscle injury in mice, M1-like macrophage depletion completely inhibited muscle regeneration, and M2-like macrophage depletion reduced the effect of tissue remodeling, respectively [21,22]. However, the immune response and temporal macrophage polarization in response to nanogels and its hydrogels have not been fully investigated. Also, high-molecular-weight HA is highly viscous with poor injectability, rendering its application in in situ regeneration difficult. Furthermore, a limited number of drug delivery substrates are known to gel in situ while stably retaining cytokines. In this study, we synthesized HA derivatives modified with cholesterol and a maleimide moiety (Chol-HA) and prepared the hydrogel via chemical crosslinking of the Chol-HA nanogel. The physicochemical and biological properties of the Chol-HA hydrogels were investigated. In particular, we focused on the macrophage response to the Chol-HA hydrogels in order to understand the immune response to the Chol-HA hydrogel and design the Chol-HA hydrogel for the wide application of HA-based hydrogels to DDS and tissue engineering. In addition, the effect of cholesterol derivative substitution on THP-1 cells, an immune cell type, adhesion to the hydrogel and secreted inflammatory cytokines was determined at a constant hyaluronan main-chain molecular weight.

## 2. Results 

### 2.1. Synthesis of Chol-HA

HA, with a molecular weight of 120 kDa, was used to synthesize Chol-HA from tetrabutylammonium hyaluronan (which was prepared as previously described [20]). Specifically, synthesis was achieved through the condensation of the cholesteryl-6-aminohexylcarbamate amine group with the HA carboxylic acid moiety, using DMT-MM. The structures of the synthesized maleimide-modified HA derivatives at different degrees of cholesterol derivative (1, 5, 10, and 15%) were confirmed via 1H NMR (Figure S1). A maleimide- characteristic peak was also identified at 6.9 ppm, and the resulting degree of substitution was determined as 15 maleimide groups per 100 HA units. 

### 2.2. Characterization of the Chol-HA Nanogel Using Dynamic Light Scattering (DLS)

The particle size associated with aggregation in solution was measured using DLS (Table 1). When dissolved in a phosphate-buffered solution (2 mg/mL, pH 7.4), Chol-HA self-aggregated into nanoparticles in water. The particle size decreased with an increase in Chol. The smallest particle size was observed at a Cholesterol moiety of 15%. Normally, HA does not form nanoparticles; however, when modified with 15% maleimide, particles as small as 180 nm were formed.

### 2.3. Preparation of the Chol-HA Hydrogel

**Figure 1:**
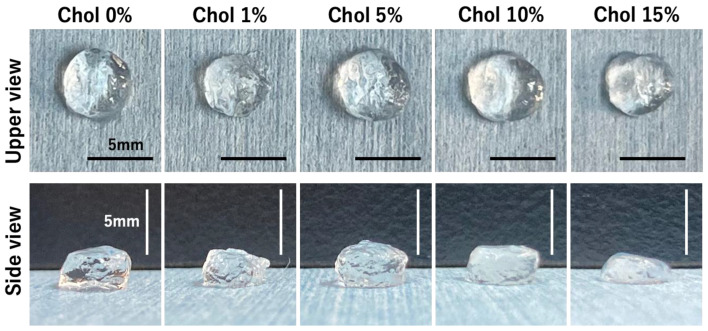
Photographs of Chol-HA hydrogels modified with different cholesterol levels. Scale bar: 5 mm.

**Figure 2:**
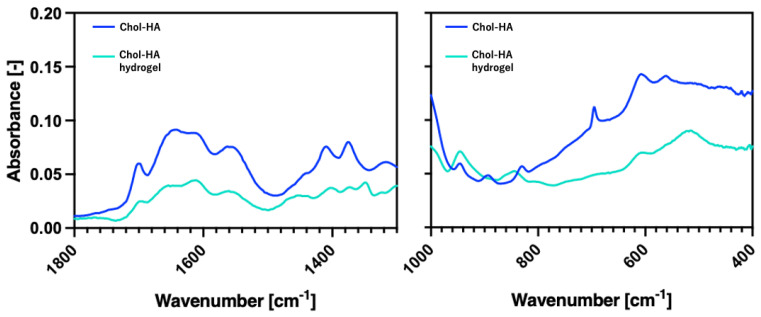
ATR-FTIR spectra of Chol-HA and Chol-HA hydrogel (5% Chol).

To prepare the hydrogels, Chol-HA nanogel and 4arm-PEGSH solutions were poured into a disk-shaped silicone rubber mold with a diameter of 6 mm and a depth of 3 mm, and then, incubated at 37 °C for 24 h. Figure 1 shows the formation of Chol-HA hydrogels at various cholesterol levels. Transparent hydrogels were obtained from all the Chol-HA samples. For the Chol-HA hydrogels with 0, 1, 5, 10% cholesterol, the gel shape was maintained when the silicone mold was removed. However, the Chol-HA hydrogel with 15% cholesterol deformed upon removal from the silicone mold. Figure 2 verifies the formation process of Chol-HA hydrogels using ATR-FTIR spectroscopy. A decrease in the peak at 1695 cm^−1^ derived from maleimide, and the disappearance of the peak at 690 cm^−1^, were observed.

**Figure 3:**
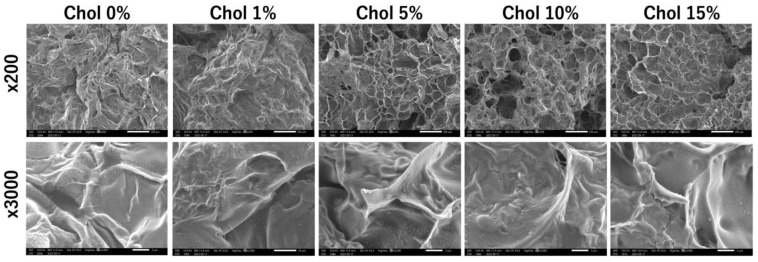
SEM images of the Chol-HA hydrogel surfaces. Scale bar: 100 µm and 1 µm.

Figure 3 shows SEM images of the Chol-HA hydrogel surfaces after freeze-drying. The 0.1% Chol-HA hydrogel exhibited a rough surface without pores. The 5, 10, and 15% Chol-HA hydrogels exhibited pores of approximately 700 µm^2^ on their surfaces (Figure 3, upper images). In addition, for all samples, dots were observed on the hydrogel (Figure 3, lower images).

### 2.4. Mechanical Properties of the Chol-HA Hydrogel 

**Figure 4:**
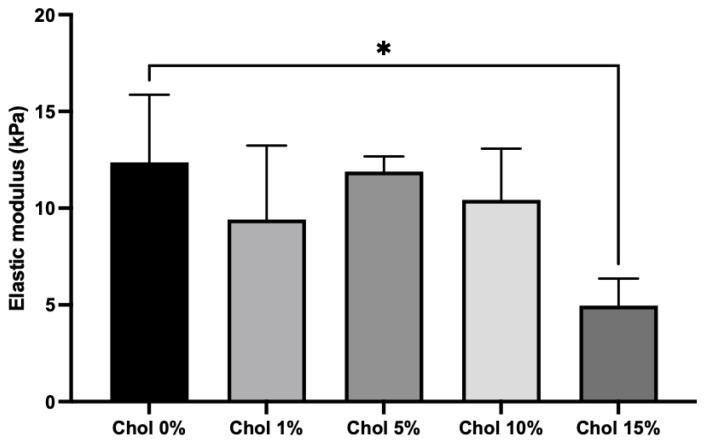
Elastic modulus of the Chol-HA hydrogels determined using compression tests (*n* = 3, * *p* < 0.05).

The mechanical properties of the Chol-HA hydrogels were evaluated using a compression test. There was no significant difference in the elastic moduli of the Chol-HA hydrogels with 0, 1, 5, and 10% cholesterol (Figure 4). However, the elasticity of the Chol-HA hydrogel with 15% cholesterol was lower than that of the Chol-HA nanogel with 0% cholesterol.

### 2.5. Cell Adhesion on the Chol-HA Hydrogel

**Figure 5:**
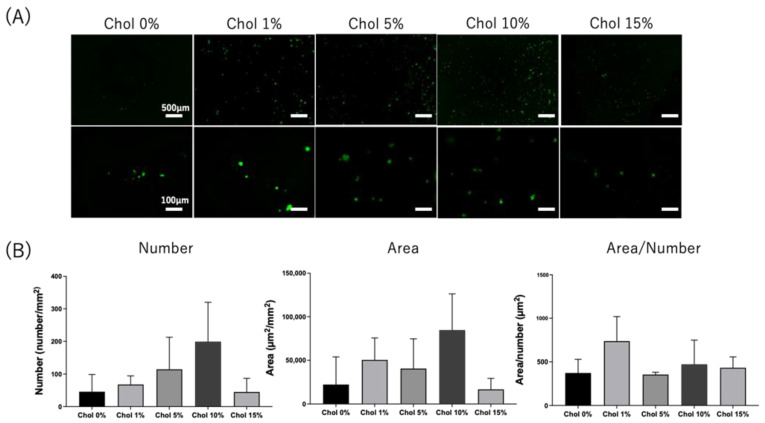
(**A**) Fluorescence image of NIH3T3 stained with calcein-AM on Chol-HA hydrogels. (**B**) Quantitative analysis of NIH3T3 adhesion on Chol-HA hydrogels, using Hybrid Cell Count software (Ver. 1.4.1.1) (*n* = 5).

First, we investigated whether NIH3T3 cells, which are fibroblasts without CD44, adhered to the Chol-HA hydrogels. NIH3T3 cells were seeded on the hydrogels and observed using fluorescence microscopy following calcein-AM staining, which stained viable cells. Spherical cells without elongation were observed on the hydrogels, irrespective of cholesterol introduction (Figure 5A). Several cells were aggregated. The number and area of adhered cells were calculated using fluorescence images (Figure 5B). There was no significant difference in the number and area of adherent cells between the Chol-HA hydrogels, irrespective of cholesterol addition. The area of cells divided by the number of cells was investigated as an index of cell aggregation. There was no significant difference in cell aggregation between Chol-HA hydrogels with various cholesterol moieties.

**Figure 6:**
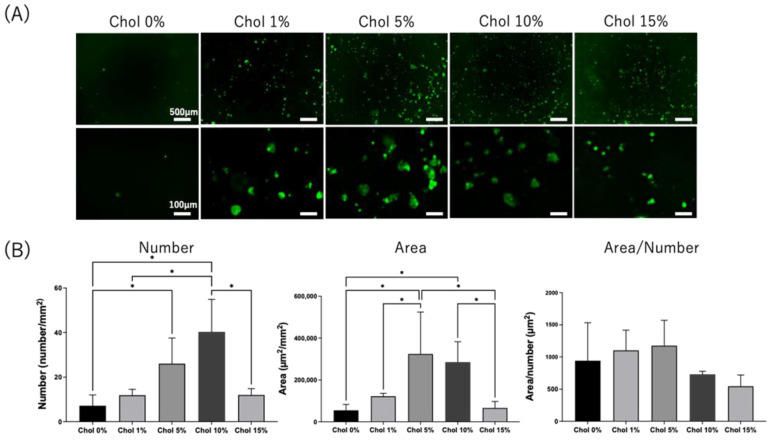
(**A**) Fluorescence image of calcein-AM-stained HiBiT-THP-1 on Chol-HA hydrogels. (**B**) Quantitative analysis of THP-1 adhesion on Chol-HA hydrogels, determined using Hybrid Cell Count software (Ver. 1.4.1.1) (*n* = 5, * *p* < 0.05).

Second, HiBiT-THP-1 cells’ adhesion to Chol-HA hydrogels was investigated. THP-1 is a monocyte CD44-positive cell line used to explore macrophage-like differentiation upon PMA stimulation. HiBiT-THP-1 cells are genetically modified with the HiBiT peptide, which is a short peptide of 11 amino acids that can bind to LgBiT and shows luminescent activity [23]. PMA-stimulated HiBiT-THP-1 cells were seeded on the hydrogels and observed using fluorescence microscopy after cultivation for 6 days and calcein-AM staining (Figure 6A). Spherical cells without elongation and aggregated cells were observed for all Chol-HA hydrogels. The cell numbers and areas of Chol-HA hydrogels with various cholesterol moieties were measured (Figure 6B). The cell numbers and area of adherent cells increased with an increase in the amount of cholesterol moiety, and higher adhesion was achieved for the hydrogels of Chol-HA with cholesterol levels of of 5 and 10%. The cell adhesion decreased in the Chol-HA hydrogels with 15% cholesterol.

### 2.6. Pro-Inflammation Response of HiBiT-THP-1 to the Chol-HA Hydrogel 

**Figure 7:**
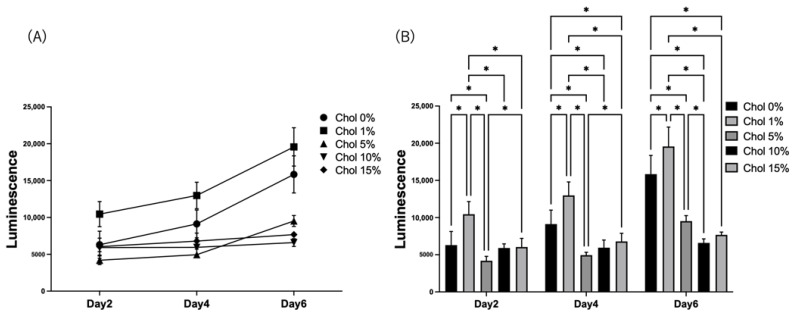
Measurement of the IL-1β secreted from HiBiT-THP-1 seeded on Chol-HA hydrogels using HiBiT assay. (**A**) Time course of IL-1β secretion. (**B**) Comparison between samples on each day (*n* = 5, * *p* < 0.05).

IL-1β is an inflammatory cytokine. The HiBiT gene was genetically inserted under the IL-1β gene for HiBiT-THP-1. Therefore, IL-1β secretion was measured via luminescence. HiBiT-THP-1 was seeded on the hydrogels, and the IL-1β secretion from HiBiT-THP-1 was measured using a HiBiT assay (Figure 7). For the Chol-HA hydrogels with 0 and 1% cholesterol, the luminescence increased over time. For the Chol-HA hydrogels with 5% cholesterol, the luminescence increased on Day 6. Constant luminescence was observed for the Chol-HA hydrogels with 10% and 15% cholesterol (Figure 7A). The luminescence of the Chol-HA hydrogels with 5%, 10%, and 15% cholesterol was lower than those with 0% and 1% (Figure 7B). 

**Figure 8:**
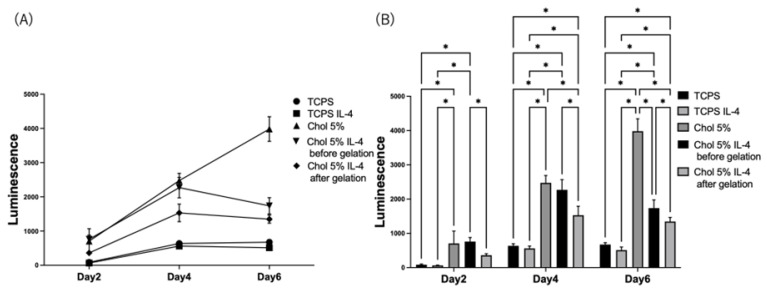
Measurement of the IL-1β secreted from THP-1 seeded on Chol-HA hydrogels, including IL-4, using HiBiT assay. (**A**) Time course of IL-1β secretion. (**B**) Daily comparison of samples (*n* = 5, * *p* < 0.05).

The effects of IL-4 encapsulation on the Chol-HA hydrogels were determined. The Chol-HA hydrogel with 5% cholesterol that exhibited a pro-inflammatory HiBiT-THP-1 cell response was investigated using the hydrogels encapsulating IL-4 before and after gelation (Figure 8). In both cases, the luminescence increased on Day 4, and then, decreased on Day 6 (Figure 8A), whereas the luminescence of the Chol-HA hydrogel with 5% and without IL-4 expression increased over time. The luminescence of the hydrogel expressing IL-4 was lower than that of the hydrogel without IL-4. Also, there was a difference in the luminescence of the hydrogel encapsulating IL-4 after and before gelation, the hydrogel encapsulating IL-4 after gelation was lower than that of the hydrogel encapsulating IL-4 before gelation (Figure 8B).

## 3. Discussion

In this study, we prepared a novel HA hydrogel through Chol-HA nanogel crosslinking and investigated its biological properties. Hyaluronic acid modified with cholesterol (modification rate: 0–15%) and maleimide (Chol-HA) was synthesized. The hydrodynamic diameter of the Chol-HA nanogel in 10 mM phosphate buffer was measured via DLS. The average particle sizes of the Chol-HA nanogels tended to decrease with an increasing degree of substitution of the cholesterol moiety. These results suggest that Chol-HA, which has 15 maleimide groups per 100 HA units, was physically crosslinked via hydrophobic interactions between the cholesteryl groups of Chol-HA to form Chol-HA nanogels [20].

The Chol-HA nanogel could crosslink with 4arm-PEGSH via Michael addition, and the hydrogel of Chol-HA nanogel was obtained. Transparent Chol-HA hydrogels were prepared with different cholesterol incorporation ratios. With the exception of the Chol-HA hydrogel with 15% cholesterol, the Chol-HA hydrogel shape was maintained upon mold removal. The elastic modulus of the Chol-HA hydrogel with 15% cholesterol was lower than that of the other hydrogels, indicating that crosslinking in the Chol-HA hydrogel did not occur sufficiently. Although we do not have a clear answer as to the mechanism, we believe that it is due to the large molecular weight between crosslinking points. Dynamic light scattering (DLS) analysis showed that the number of associations was highest and particle size was smallest at 15% Chol incorporation, suggesting that the distance between crosslinks of nanogels was larger at Chol 15%, or that the distance between crosslinking points was larger and the elastic modulus was low because the crosslinking groups were unreacted. 

Pore sizes were observed on the hydrogel surfaces using SEM. For Chol-HA hydrogels with 5, 10, and 15% cholesterol, pores were observed on the gel surface. Pores are formed during the freezing process. The hydration of Chol-HA with 0 and 1% cholesterol was different to that of hydrogels with 5, 10 and 15% cholesterol. In addition, dots were observed on the Chol-HA hydrogel, irrespective of the cholesterol incorporation ratio. The size of the dots decreased with increasing cholesterol, and the size of the Chol-HA nanogel before gelation, as measured using DLS, decreased with increasing cholesterol. From these results, it is presumed that the Chol-HA nanogels were observed as dots in the SEM images, indicating that the hydrogel was formed due to the Chol-HA nanogels crosslinking. 

The adhesion of NIH3T3 and THP-1 cells to the Chol-HA hydrogels was also investigated. Few NIH3T3 cells adhered to all hydrogels, whereas the adhesion of HiBiT-THP-1 increased with an increase in cholesterol. CD44 is an HA receptor [24]. NIH3T3 cells were CD44-negative, and HiBiT-THP-1 cells were CD44-positive cells; thus, HiBiT-THP-1 cells could adhere to the Chol-HA hydrogel [25]. The shape of the HiBiT-THP-1-adherent cells was spherical; spread and elongation of the cells was not observed on the Chol-HA hydrogels. THP-1 cells are monocytes that differentiate into macrophage-like cells via PMA stimulation [26]. In this study, HiBiT-THP-1 cells were seeded after PMA stimulation to investigate the macrophage response to Chol-HA hydrogels. For the control of TCPS, spread and elongated cells were observed, which are typical adhesion formations in integrin-adhesion protein interactions. Thus, the adhesion of HiBiT-THP-1 cells to the Chol-HA hydrogel, with a spherical formation, was probably mediated by the CD44-hyarulonic acid interaction. Because the interaction between CD44 and HA was weaker than that of the integrin-mediated cell adhesion, the ratio of adhered cells to seeded cells was approximately 1.2%. Interestingly, the adhesion of HiBiT-THP-1 cells increased with increasing cholesterol introduction into HA. The cells contacted and bonded via CD44-hyaluronic acid interaction and may have interacted with the cholesterol assembly in the nanogel, resulting in the maintenance of cellular adhesion on the hydrogels of Chol-HA. This mechanism remains unknown and should be investigated in detail in future studies. 

The pro-inflammatory macrophage response to the Chol-HA hydrogels was investigated using HiBiT-THP-1; the HiBiT gene was genetically inserted under the IL-1b gene [23]. For this system, the IL-1β secretion could be measured via luminescence. The luminescence was strongly dependent on cholesterol introduction and was inhibited at higher cholesterol levels in the hydrogels. As described above, HiBiT-THP-1 cells adhered to CD44. The binding of CD44 to macrophages stimulates pro- and anti-inflammatory responses in macrophages [2]. In this case, the pro-inflammatory macrophage response bound through the CD44–hyaluronic acid interaction was inhibited on the hydrogels of Chol-HA because the luminescence decreased with increasing cell adhesion. However, for the hydrogel with 15% cholesterol, low luminescence was exhibited. However, the cell adhesion was low, and was similar to that on hydrogels with 0 and 1% cholesterol. Pro- and anti-inflammatory stimulation via the CD44–hyaluronic acid interaction was not sufficient because the Chol-HA binding site was decreased by cholesterol modification. Also, it may be considered that the lowest elastic modulus of the Chol-HA hydrogel with 15% cholesterol induced low inflammatory activation [27,28], or the cholesterol in the nanogel affected the inhibition of pro-inflammatory and enhancement of anti-inflammatory reactions, although the mechanism is still unknown.

Chol-HA hydrogels encapsulating IL-4, an anti-inflammatory cytokine, were prepared to induce an anti-inflammatory response in macrophages [29,30,31]. Compared to the hydrogel of Chol-HA without IL-4, IL-1β secretion was inhibited and high inhibition of IL-1β secretion was achieved on Day 6. Interestingly, the Chol-HA hydrogel, in which IL-4 was encapsulated after gelation, inhibited the proinflammatory response of macrophages. It was suggested that IL-4 was released and affected macrophage response. Further investigation for the release profiles of the hydrogels before and after gelation is needed. 

## 4. Conclusions

In the present study, we successfully synthesized an HA-modified hydrogel using cholesterol and maleimide. The Chol-HA hydrogel was obtained via chemical crosslinking of the Michael addition reaction (between the maleimide and thiol groups of 4arm-PEGSH). It was found that Chol-HA hydrogels with higher cholesterol modification inhibited IL-1β secretion, which is one pro-inflammatory response of macrophage-like THP-1 cells. Moreover, IL-4 encapsulated in the Chol-HA hydrogel enhanced the inhibition of inflammation in THP-1 cells. These results provide useful insights into the biomedical applications of hydrogels. 

## 5. Materials and Methods 

### 5.1. Materials

HA (average molecular weight:120 kDa) was purchased from Bloomage Biotechnology Co., Ltd. (Tokyo, Japan). Cation exchange resin (Dowex^®^ 50WX-8-400) was purchased from Sigma-Aldrich (Tokyo, Japan). Cholesteryl chloroformate, N-Boc-1,6-hexanediamine, and N-(5-Aminopentyl)maleimide hydrochloride were purchased from Tokyo Chemical Industry Co., Ltd. (Tokyo, Japan). The hydrochloride 4-(4,6-Dimethoxy-1,3,5-triazin-2-yl)-4-methylmorpholinium chloride (DMT-MM) was purchased from Kokusan Chemical Co., Ltd. (Tokyo, Japan), and 4arm-PEGSH (molar weight = 1 × 104 g/mol) from the NOF Corporation (Tokyo, Japan). NIH3T3 and THP-1 cells were purchased from the RIKEN Bioresource Center. DMEM, RPMI-1640 medium, phosphate-buffered saline (PBS), penicillin/streptomycin solution (×100), and Phorbol 12-Myristate 13-Acetate were purchased from FUJIFILM Wako Pure Chemical Corp. (Tokyo, Japan). Interleukin 4 (IL-4) was purchased from BioLegend (San Diego, CA, USA). Fetal bovine serum (FBS) was purchased from Sigma-Aldrich. Calcein-AM was purchased from Dojindo Laboratories (Kumamoto, Japan). The Nano-Glo HiBiT Extracellular Detection System was purchased from Promega Japan Co. (Tokyo, Japan).

### 5.2. Preparation of Hyaluronic Acid Modified with Cholesterol and Maleimide (Chol-HA)

HA was modified with cholesterol and maleimide derivatives, in solution, to create a new hydrogel precursor. Cholesteryl-6-aminohexylcarbamate and hyaluronic acid tetra-n-butylammonium salt (HA-TBA) were prepared as previously described [15]. HA was converted, by reacting a cation-exchange resin with N-(5-Aminopentyl)maleimide hydrochloride using DMT-MM as a condensing agent, to HA-TBA. HA-TBA was fully dissolved in dimethyl sulfoxide (DMSO, 1% w/v), and then, N-(5-Aminopentyl)maleimide-containing DMSO was added and stirred for 5 min at room temperature. Subsequently, DMT-MM was added to the mixture and stirred overnight at room temperature. The feed molar ratio was 100:20:24 (glucuronic acid of HA:DMT-MM: N-(5-Aminopentyl)maleimide hydrochloride; x = 20). Then, cholesteryl-6-aminohexylcarbamate was similarly prepared. The feed molar ratio was 100:x:1.2 × (glucuronic acid of HA:DMT-MM:cholesteryl-6-aminohexylcarbamate; x = 1.2, 5, 10, 16, and 22). The HA product was purified via dialysis at 12–14 kDa against DMSO, 0.150M NaCl, and distilled water. The purified HA solution was filtered through a 0.22 µm membrane filter, and lyophilized until dry. For the NMR analysis, Chol-HA was dissolved in 0.02N DCl/DMSO. 1H-NMR spectra were obtained using a 400 MHz NMR instrument (JNM-ECS400).

### 5.3. Dynamic Light Scattering Measurement of Chol-HA Nanogel

The Chol-HA solution in 10 mM phosphate buffer (pH 7.4) was characterized via dynamic light scattering (DLS) analysis using an ELSZ-2000 instrument (Otsuka Electronics). Autocorrelation functions were calculated using the cumulant method. The hydrodynamic diameter of Chol-HA was analyzed using the Stokes–Einstein equation.

### 5.4. Preparation of Chol-HA Nanogel-Based Hydrogel (Chol-HA Hydrogel)

Chol-HA hydrogel was prepared through crosslinking, via a Michael addition reaction between the maleimide and thiol groups. Briefly, the Chol-HA (10 mg/mL) and 4arm-PEGSH (20 mg/mL) were dissolved separately in 10 mM phosphate buffer (pH 7.4). Subsequently, the two precursor solutions were poured into disk-shaped silicone rubber molds (mold diameter and depth: 8 mm and 1.5 mm, and 6 mm and 3 mm), which were placed on a polytetrafluoroethylene membrane and cooled to 5 °C. Next, a silicone coversheet was used to cover the mold, which was incubated at 37 °C for 30 min. After incubation, the samples were transferred into well plates. The molar ratio of the maleimide to thiol groups was 1.1:1.

### 5.5. Total Reflectance–Fourier Transform Infrared (ATR-FTIR) Spectroscopic Analysis

ATR-FTIR spectra were recorded using an ATR-FTIR spectrometer (Perkin Elmer, Waltham, MA, USA). The spectra were obtained in the range of 4000–400 cm^−1^ and at a 4 cm^−1^ resolution with 8 co-added scans per spectrum. The absorption intensities under the spectra were integrated at wavenumbers of 1800–1300 and 1000–400 cm^−1^, which represent the regions of maleimide and alkene C-H, respectively. The freeze-dried samples of Chol-HA and Chol-HA hydrogel were subjected to IR measurements.

### 5.6. Encapsulation of IL-4 in the Chol-HA Hydrogel 

Before and after gelation, IL-4 was encapsulated in the Chol-HA hydrogels. Before gelation, the Chol-HA (10 mg/mL) nanogel solution was mixed with the IL-4 (2 µg/mL) solution and incubated at 4 °C for 24 h, and then, gelatinized at 37 °C for 30 min. Next, IL-4 was added to the Chol-HA nanogel solution prior to gelation such that IL-4 was 20 ng/mL after gelation. After gelation, the Chol-HA nanogel solution was gelatinized at 37 °C for 30 min and the hydrogel was soaked in the IL-4 (20 ng/mL) solution at 4 °C for 24 h.

### 5.7. Scanning Electron Microscope (SEM) Observation

The Chol-HA hydrogels were freeze-dried using a freeze-dryer (FDU-100, TOKYO RIKAKIKI Co., Ltd., Tokyo, Japan). The hydrogels were coated in PI and observed using a scanning microscope (JSM-IT200, JEOL Corp., Tokyo, Japan), at 10 kV.

### 5.8. Compression Test

The mechanical properties of the Chol-HA hydrogels were evaluated via compression tests using a RHEONER II (Creep meter RE2-330058, YAMADEN Co., Ltd., Tokyo, Japan). Chol-HA hydrogel disks (concentration: 7 mg/mL, diameter: 6 mm, and height: 3 mm) were used. The Chol-HA hydrogels were placed on a plate and loaded to 20% strain with a maximum load of 0.2 N and a load speed of 0.05 mm/s.

### 5.9. Cell Culture

NIH3T3 cells were maintained in DMEM medium and supplemented with 10% FBS and 1× penicillin/streptomycin at 37 °C and 5% CO_2_. HiBiT-tagged THP-1 cells, in which the HiBiT gene was inserted into IL-1β, were generated using the CRISPR/Cas9 system, as previously described [23]. HiBiT-THP-1 cells were maintained in PRMI-1640 medium supplemented with 10% FBS, 1× penicillin/streptomycin/L-glutamine, 10 mM HEPES, and 1 mM sodium pyruvate at 37 °C and 5% CO_2_. HiBiT-THP-1 monocytes were differentiated in macrophage-like cells using RPMI-1640 medium (containing 10% FBS, 1× penicillin/streptomycin and PMA (320 µM)).

### 5.10. Cell Culture on the Chol-HA Hydrogel

Chol-HA hydrogels were added to 48-well plates. The NIH3T3 cell suspension was prepared in EMEM (containing 10% FBS and 1× penicillin/streptomycin) at 1.14 × 10^4^ cells/mL. The cell suspension (0.5 mL) was seeded in each plate well and the plate was incubated at 37 °C and 5% CO_2_ for 1, 3, and 5 days. The HiBiT-THP-1 cell suspension was prepared in RPMI-1640 (containing 10% FBS and PMA (320 µM)) and seeded (4.5 × 10^5^ cells/mL) in each well, and the plate was incubated at 37 °C and 5% CO_2_ for 1, 3, and 5 days.

### 5.11. Fluorescent Microscopic Observation of Cells on the Chol-HA Hydrogel

The cells on the hydrogels were washed with PBS and stained with Calcein-AM. The hydrogels were then transferred to another plate and observed under a fluorescence microscope (BZ-X710; Keyence Corp., Osaka, Japan). Using fluorescence images of cells stained with calcein-AM on the hydrogel, the cell number and area were measured using Hybrid Cell Count software (BZ-X710, Keyence, Corp., Osaka, Japan). Although single and aggregated cells were observed in the fluorescence images, the aggregated cells were counted as one cell without cell separation. The cell number and area were represented as the number per mm^2^ and cell area (mm^2^)/sample area (mm^2^). The ratio of the cell area to the cell number was calculated as an index of cell aggregation.

### 5.12. HiBiT Assay

The HiBiT-THP-1 cells were seeded onto the Chol-HA hydrogel. Then, 10 µL of supernatant was collected after 2, 4, and 6 days of incubation, diluted with 90 µL PRMI-1640 medium (supplemented with 10% FBS, 1× penicillin/streptomycin/L-glutamine, 10 mM HEPES, and 1 mM sodium pyruvate), and mixed with 50 µL of a mixture of buffer, substrate, and LgBiT (100:2:1) using a Nano-Glo^®^ HiBiT Extracellular Detection System. Luminescence was measured using a multispectrometer (Cytation, BioTek Japan, Tokyo, Japan).

### 5.13. Statical Analysis

The results are expressed as the mean standard deviation. Statistical analyses were performed using one-way analysis of variance (ANOVA). Samples were compared using Tukey’s multiple comparison test. Statistical significance was set such that *p* < 0.05 was considered significant. Each experiment was performed five times, except for the compression test, with a trial number of 3.

## Figures and Tables

**Table 1:** DLS measurement of Chol-HA nanogel.

Cholesterol Incorporation (%)	Size (nm)	PDI (-)
0	178 ± 4.2	0.525 ± 0.030
1	146 ± 2.4	0.467 ± 0.003
5	106 ± 1.4	0.398 ± 0.001
10	73 ± 1.8	0.335 ± 0.003
15	37 ± 0.3	0.291 ± 0.005

## Data Availability

Not applicable.

## Supplementary Materials

The following supporting information can be downloaded at: https://www.mdpi.com/article/10.3390/gels9110866/s1, Figure S1: NMR charts of Chol-HA with cholesterol of (A) 0%, (B) 1%, (C) 5%, (D) 10%, and (E) 15%.

## Abbreviations

Chol	Cholesterol
HA	Hyaluronic acid
HA-TBA	Hyaluronic acid tetra-n-butylammonium salt
DMT-MM	Hydrochloride 4-(4,6-Dimethoxy-1,3,5-triazin-2-yl)-4-methylmorpholinium chloride
FBS	Fetal bovine serum
TCPS	Tissue culture polystyrene
PMA	Phorbol 12-Myristate 13-Acetate
LPS	Lipopolysaccharide
IL-4	Interleukin 4
