# Supplementary material for: Preparation of Cholesterol-Modified Hyaluronic Acid Nanogel-Based Hydrogel and the Inflammatory Evaluation Using Macrophage-like Cells
Source: Gels. 2023 Oct 31;9(11):866. doi: 10.3390/gels9110866 (PMC10671248; doi:10.3390/gels9110866)
Supplement: Supplementary file 1 [file gels-09-00866-s001.zip › gels-2665074-supplementary.pdf]

gels

Supplementary Materials

Preparation of Cholesterol-Modified Hyaluronic Acid Nanogel-Based Hydrogel and the Inflammatory Evaluation Using Macrophage-like Cells

Kohei Yabuuchi [1,2], Mika Suzuki [2], Chen Liang [2], Yoshihide Hashimoto [2], Tsuyoshi Kimura [2,*], Kazunari Akiyoshi [3] and Akio Kishida [2,*]

1 New Product Development Office, R&D Group, Healthcare Materials Division, Life Innovation SBU, Asahi Kasei Co., Chiyoda-ku, Tokyo 100-0006, Japan
2 Institute of Biomaterials and Bioengineering, Tokyo Medical and Dental University, 2-3-10 Kanda-surugadai, Chiyoda-ku, Tokyo 101-0062, Japan
3 Department of Immunology, Graduate School of Medicine, Kyoto University, Yoshida-Konoe-cho, Sakyo-ku, Kyoto 606-8501, Japan
* Correspondence: kimurat.mbme@tmd.ac.jp (T.K.); kishida.mbme@tmd.ac.jp (A.K.); Tel.: +81-3-5280-8028 (A.K.)

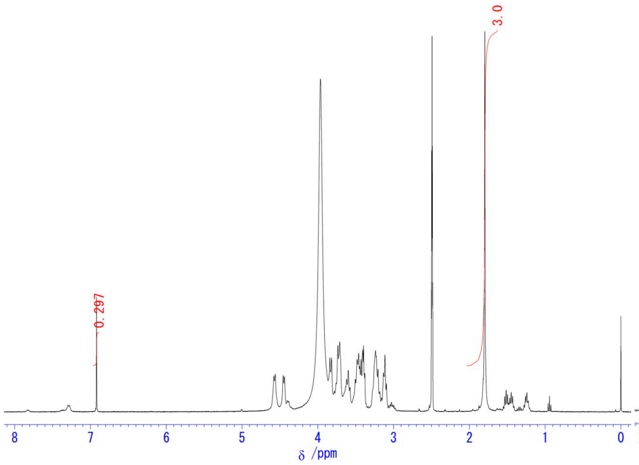

(**A**)

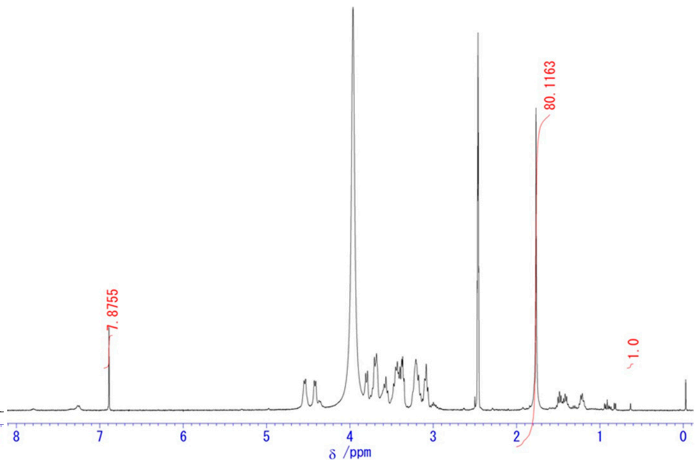

(**B**)

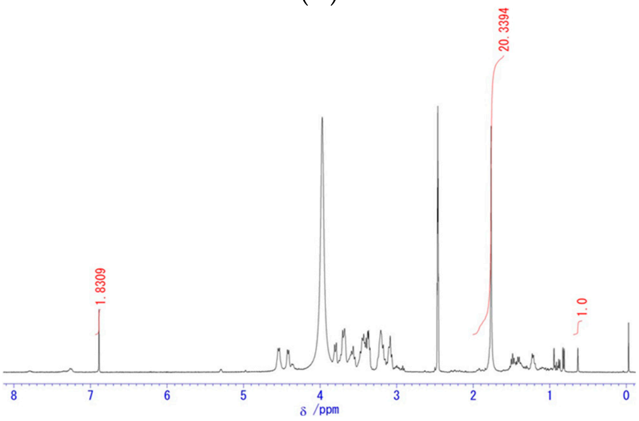

(**C**)

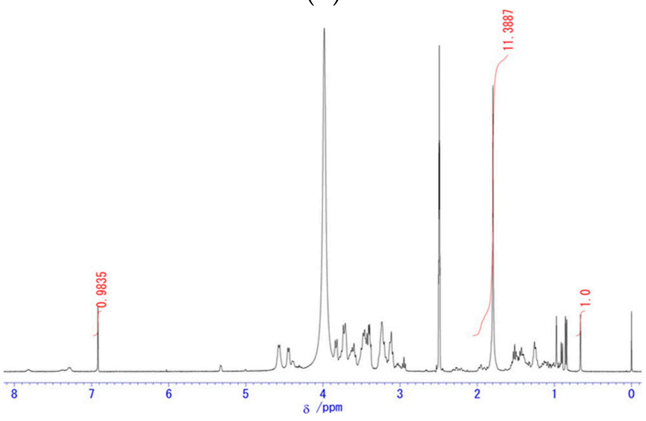

(**D**)

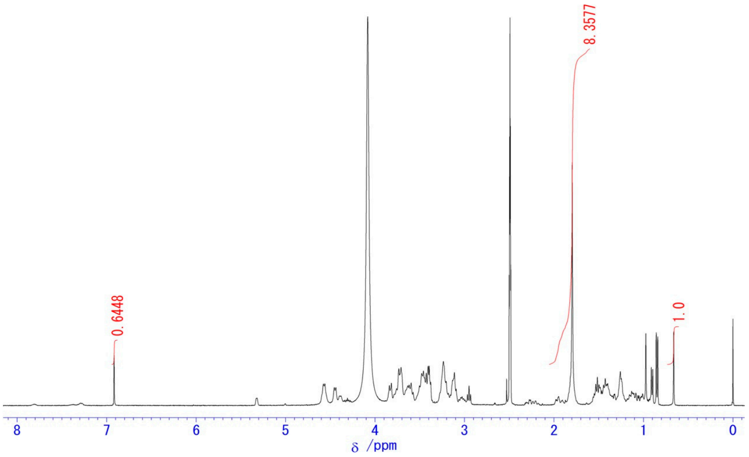

(**E**)

Figure S1. NMR charts of Chol−HA with cholesterol of (**A**) 0%, (**B**) 1%, (**C**) 5%, (**D**) 10%, and (**E**) 15%.
